# The antimicrobial effect of a novel peptide LL-1 on *Escherichia coli* by increasing membrane permeability

**DOI:** 10.1186/s12866-022-02621-y

**Published:** 2022-09-19

**Authors:** Lingling Zhou, Kaiqi Lian, Mengting Wang, Xueyi Jing, Yuanchen Zhang, Jinling Cao

**Affiliations:** 1 College of Veterinary Medicine, Shanxi Agricultural University, Taigu, 030801 Shanxi, People’s Republic of China; 2 College of Biology and Food Engineering, Anyang Institute of Technology, Anyang, 455000 Henan People’s Republic of China; 3 Henan Joint International Research Laboratory of Veterinary Biologics Research and Application, Anyang, 455000 Henan People’s Republic of China; 4 Taihang Mountain Forest Pests Observation and Research Station of Henan Province, Linzhou, 456550 Henan People’s Republic of China; 5 College of Food Science and Technology, Shanxi Agricultural University, Taigu, 030801 Shanxi, People’s Republic of China

**Keywords:** Antibacterial effect, Antimicrobial peptide, β-galactosidase, *Escherichia coli*, Membrane permeability

## Abstract

**Background:**

The widespread use of antibiotics has led to the emergence of many drug-resistant strains; thus, the development of new antibacterial drugs is essential with antimicrobial peptides becoming the focus of research. This study assessed the antibacterial effect of a novel antimicrobial peptide, named LL-1 on *Escherichia coli* (*E.coli*) by determining the minimum inhibitory concentration (MIC) and the antibacterial curve. The interaction between LL-1 and *E. coli* DNA was then detected by nucleic acid gel electrophoresis. The effect of LL-1 on the *E. coli* cell membrane was assessed by detecting the leakage of β-galactosidase, nucleic acid and protein. The influence of LL-1 on the intracellular ATP of *E. coli* was analysed by determining the concentration of intracellular ATP. Finally, the bacteria and colonies of *E. coli* treated with LL-1 were observed using scanning and transmission electron microscopy.

**Results:**

The results suggested that the MIC value was 3.125 µg/ml, and the antibacterial effect was dose-dependent. LL-1 dose-dependently combined with *E. coli* DNA. LL-1 resulted in the leakage of intracellular β-galactosidase, nucleic acid and protein, and decreased intracellular ATP concentrations of *E. coli*. Two MIC of LL-1 caused *E. coli* to shrink, resulting in a rough surface, plasmolysis, and bacterial adhesion.

**Conclusion:**

This study indicated that LL-1 had a good bactericidal effect on *E. coli* by mainly increasing the permeability of the cell membrane, leading to leakage of the intracellular content. This will lay the foundation for an in-depth study on the antibacterial mechanism of LL-1 against *E. coli* and its clinical application.

**Supplementary Information:**

The online version contains supplementary material available at 10.1186/s12866-022-02621-y.

## Background

The abuse of antibiotics in aquaculture has led to serious drug resistance issues [1, 2]. The continuous emergence of super drug-resistant bacteria has caused a series of problems, such as the imbalance of flora in the body and environmental pollution, which has resulted in a significant threat to global public safety [3]. Thus, it is urgent to reduce the use of antibiotics and develop the alternatives to antibiotics [4–6]. Antibacterial peptides are a special class of small molecules with biological activity and are considered as an ideal alternative to antibiotics due to their characteristics of small molecular weight, low toxicity, broad antibacterial spectrum, wide variety of sources, unique antibacterial mechanism, and few side effects [7–10]. Antibacterial peptides are derived from a wide range of sources and can be isolated from plants, insects, mammals, amphibians and even bacteria [11]. Antibacterial peptides are important part of the biological innate immune system and are induced by immunity to resist infection by external pathogens [12, 13]. They constitute the natural protective barrier of the organism and can directly kill various pathogenic microorganisms or indirectly resist the invasion of viruses, fungi, bacteria, parasites and other pathogens by regulating the body’s immune system [14].

There are many ways in which antimicrobial peptides exert their antibacterial effects [15, 16]. According to the different components of bacteria acted by antimicrobial peptides, they can be divided into cell walls, cell membranes and intracellular substances. When antimicrobial peptide interacts with bacteria, it first contacts the cell wall, which may change the permeability of the cell wall, so that the bacteria cannot maintain the complete cell shape, in order to achieve the antibacterial effect. The damage caused by antimicrobial peptides to bacterial cell walls depends not only on the charge and hydrophobicity of antimicrobial peptides, but also on the amount of bacteria [17]. It is a common antibacterial method that antimicrobial peptides disrupt bacterial cell membranes. Antimicrobial peptides fuse with lipid membranes through molecular amphiphilicity to form ionic pore channels. Different pore-forming methods are mainly caused by different physical and chemical properties [18]. Antimicrobial peptides can also act on intracellular substances after passing through the cell membrane. When antimicrobial peptides enter bacteria, they may combine with bacterial nucleic acid, which results in bacteria losing their normal replication and transcription functions causing abnormal expression of the proteins required for the growth and development of bacteria. In addition, antimicrobial peptide may interact with bacterial protein, resulting in changes of protein structure, inactivation of many bacterial enzymes, and disorder of metabolism, causing bacteria to die [19–21].

*Escherichia coli* (*E. coli*) is an important bacterium and causes a wide range of infections and great harm [22]. It has resulted in irreversible economic losses to human health, animal husbandry, and food safety [23]. At present, there are a number of resistant strains of *E. coli*, and it is particularly important to develop new drugs against *E. coli*. LL-1 is a novel antimicrobial peptide isolated from *Dichocrocis punctiferalis* (Guenée) and identified by our laboratory through transcriptome sequencing and sequence alignment analysis. In this study we evaluated the antibacterial effect and mode of action of LL-1 against *E. coli*.

## Materials and methods

### Materials

The *E. coli* strain ATCC25922 was preserved by the Henan International Joint Laboratory for the Development and Application of Veterinary Biological Products (Anyang, China). Antimicrobial peptide LL-1 (EPRWKGWKKIEKAVRRVRDGIIKAGPAVAVVGQVQGIAG) was synthesized and purified by Sangon Biotech (Shanghai) Co., Ltd., China. The Ezup Column Bacteria Genomic DNA Purification Kit was purchased from Sangon Biotech. DL-15000 DNA Marker was purchased from Takara Biomedical Technology (Beijing) Co., Ltd., China. O-Nitrophenyl-β-D-Galactopyranoside (ONPG) and the ATP Assay Kit were purchased from Beytime (Shanghai, China). Electron microscope fixative solution was purchased from Wuhan Sevier Biotechnology Co., Ltd., China.

### Characterizations of the LL-1 peptide

To study the LL-1 peptide, we predicted its physicochemical characteristics through the ExPASy Bioinformatics Resource Portal (http://www.expasy.org/tools/) [24].

Additionally, the hemolytic activity of LL-1 was determined using red blood cells from BALB/c mice. Blood containing anticoagulants collected from mice was washed, cells counted, and diluted as previously reported by our laboratory [25]. The red blood cell suspension was mixed with different concentrations of LL-1 (final concentrations: 50, 100, 150, 200, and 250 μg/mL). These mixtures were co-incubated at 37 °C for 1 h, and then centrifuged at 3,000 g for 10 min. The obtained supernatants were used to determine the absorbance at 405 nm. The hemolysis ratio was calculated using the following formula: hemolysis ratio = (A_405peptide_—A_405PBS_)/(A_405Triton_—A_405PBS_) × 100%.

The cytotoxicity of LL-1 was determined in porcine kidney-15 (PK-15) cells through a CCK-8 cell counting kit (Vazyme, Nanjing, China). PK-15 cells were counted, diluted and inoculated into 96-well cell-culture plates, and then cultured at 37 °C for 24 h as previously reported by our laboratory [25]. Different concentrations of LL-1 were added to the 96-well cell-culture plates at final concentrations of 50, 100, 150, 200, and 250 μg/mL, respectively. The 96-well cell-culture plates were further incubated at 37 °C for 12 h, and then 10 μL of CCK-8 reagent was added to each well. After incubation at 37 °C for 1 h, the absorbance at 450 nm was detected by an automatic multi-function microplate reader (Synergy H1, BioTek, USA).

### The minimum inhibitory concentration (MIC) of LL-1 against *E. coli*

*E. coli* was cultured in the sterile LB medium for 12 h and centrifuged for 2 min at 6,000 g at room temperature to collect the bacterial pellet, which was diluted with sterile LB medium to 2 × 10^6^ CFU/mL. LL-1 was diluted with phosphate-buffered saline (PBS) to 200 µg/mL. The MIC of LL-1 against *E. coli* was detected by double dilution method as previously described [25]. Kanamycin (200 µg/mL) was used as a positive control and PBS was used as a negative control. After treatment for 16 h, resazurin was used as an indicator, and 10 µL of 6 mM resazurin was added to each well. Following incubation at 37℃ for 3 h, the color change in each well was observe.

### Effects of LL-1 on *E. coli* growth

*E. coli* in the logarithmic growth phase was inoculated into sterile LB liquid medium at the volume ratio of 1:100. *E. coli* solutions were divided into four groups. LL-1 was added into four groups with a final concentration of 0, 1, 4, and 8 MIC LL-1, respectively. The four bacterial solutions were incubated at 37℃ with shaking at 200 rpm. Next, 100 µL of bacterial solution was removed from each group after 0, 2, 4, 6, 8, 10, and 12 h of incubation and placed in 96-well microtiter plates. The absorbance values at 600 nm were detected with an automatic multi-function microplate reader (Synergy H1, BioTek, USA). Each experiment was repeated three times.

### Effects of LL-1 on the permeability of *E. coli* cell membrane

*E. coli* in the logarithmic growth phase was centrifuged at 6,000 g and room temperature. The bacterial cells obtained were collected, washed three times with sterile PBS, resuspended and divided into five samples. LL-1 was added to the each sample with the final concentration of 0, 1, 4, and 8 MIC, respectively, and Triton X-100 served as the positive control with the final concentration of 0.3% (v/v). All samples were placed in a constant temperature water bath at 37℃ for 1 h, and then centrifuged at 6,000 g for 5 min. Then, 100 µL of the supernatant was removed and placed in a 96-well plate, mixed with 4.5 µL of ONPG at the concentration of 3 mmol/L, and then incubated at 37℃ for 30 min. The absorbance value at 420 nm was measured with an automatic multi-function microplate reader (Synergy H1, BioTek, USA). Each experiment was repeated three times.

Using the same method, the *E. coli* solutions were divided into four samples. LL-1 was added to each sample with the final concentration of 0, 1, 4, and 8 MIC, respectively. All samples were placed in a constant temperature water bath at 37℃. Then, 200 µL of bacterial solution was removed from each tube after 0, 1, 2, 3, 4, 5, and 6 h of incubation and centrifuged at 6,000 g and 4℃ for 5 min. The supernatant was removed and the absorbance was determined at 260 nm and 280 nm using a NanoDrop 2000 spectrophotometer (Thermo Fisher Scientific, USA), respectively. Three replicates were prepared for each sample.

### Binding of LL-1 to *E. coli* DNA

*E. coli* in the logarithmic growth phase was centrifuged at 6,000 g and room temperature to collect the bacterial cells. The *E. coli* genomic DNA was extracted according to the Ezup Column Bacteria Genomic DNA Purification Kit instructions, and the DNA concentration was determined using an ultra-micro spectrophotometer (NanoDrop 2000, Thermo Scientific, USA). Seven samples with final DNA concentration of 25 ng/µL in 20 µL system were then prepared according to a mass ratio of LL-1 to *E. coli* DNA of 0, 0.25, 0.5, 1, 2.5, 5 and 10, respectively. The samples were gently inverted to allow mixing and then placed in a constant temperature water bath at 37℃ for 30 min. The mixed solution was analysed by nucleic acid gel electrophoresis and observed using a nucleic acid gel imager (G:BOX, Syngene, UK).

### Effects of LL-1 on the intracellular ATP of *E. coli*

*E. coli* in the logarithmic growth phase was centrifuged at 6,000 g and room temperature. The bacterial cells were collected, washed three times with sterile PBS, resuspended and divided into four samples. LL-1 was added to a final concentration of 0, 1, 4, and 8 MIC, respectively. All samples were placed in a constant temperature water bath at 37℃ for 1 h, and then centrifuged at 6,000 g for 5 min to collect the bacterial cells. The concentration of ATP in each sample was determined according to the instructions of the ATP Assay Kit. Each experiment was repeated three times.

### Effects of LL-1 on *E. coli* observed by scanning and transmission electron microscopy

*E. coli* in the logarithmic growth phase was centrifuged at 6,000 g and room temperature to collect the bacterial cells, which were washed three times with sterile PBS, resuspended and divided into four samples. LL-1 was added into two samples with the final concentration of 2 MIC, while PBS was added to the other samples as the negative controls. All samples were placed in a constant temperature water bath at 37℃ for 1 h, and then centrifuged at 6,000 g for 5 min to collect the bacterial cells, which were washed three times with sterile PBS and resuspended in electron microscopy fixative solution. The samples were fixed at room temperature for 2 h, and sent to Wuhan Sevier Biotechnology Co., Ltd. Two groups of samples (PBS and LL-1 treated groups) were fixed by 1% (v/v) osmic acid for 2 h and dehydrated by different concentrations of ethanol and isoamyl acetate at 4 °C. The samples were dried, then closely attached to the conductive carbon film double-sided tape and placed on the sample stage of the ion sputtering instrument for gold spraying for approximately 30 s, observed, and images were obtained by scanning electron microscopy (Regulus 8100, Hitachi, Japan).

The another samples were pre-embedded in 1% agarose gel, and then fixed with 1% (v/v) osmic acid for 2 h at room temperature. The samples were dehydrated by different concentrations of ethanol and acetone at room temperature, permeabilized with acetone embedding reagents, and then polymerized in an oven at 60 °C for 48 h. The samples were placed in a microtome and 60–80 nm ultra-thin sections were obtained through a 150-mesh Fanghua film copper mesh. The copper mesh was dyed in 2% uranyl acetate saturated alcohol solution for 8 min in the dark, washed with 70% alcohol three times and then ultrapure water three times, dyed by 2.6% lead citrate solution in the absence of carbon dioxide for 8 min, washed with ultrapure water three times, and then slightly dried with filter paper. The copper mesh slices were placed in a copper mesh box to dry overnight at room temperature, observed and images obtained by transmission electron microscopy (HT7700, Hitachi, Japan). The bacterial death rate was evoluated by Image-Pro Plus 6.0 software (Media Cybernetics, USA).

### Effects of LL-1 on *E. coli* determined by PI staining experiment

*E. coli* in the logarithmic growth phase was centrifuged at 6,000 g and room temperature to collect the bacterial cells, which were washed three times with sterile PBS, resuspended and divided into five groups, and each group had 1 mL of bacterial solution. LL-1 was added to four groups at final concentration of 0, 1, 4, and 8 MIC, respectively. Triton X-100 served as the positive control with the final concentration of 0.3% (v/v). All of samples were gently inverted to allow mixing, and then placed in a constant temperature water bath at 37℃ for 1 h. Then, these samples were centrifuged at 10,000 g for 1 min. The supernatants were discarded, and the bacterial precipitations were resuspended by sterile PBS. PI was added to the bacterial solution at a final concentration of 10 nmol/L, which was gently inverted to allow mixing and incubated in the dark at 37℃ for 30 min. The fluorescence intensity was measured using a multi-function microplate reader (Synergy H1, BioTek, USA) at the excitation wavelength of 535 nm and the emission wavelength of 615 nm.

### Statistical analysis

Each experiment was repeated three times, and the results are presented as the mean and standard deviation. Data analysis and graphs were performed using GraphPad Prism software (version 6.0, La Jolla, CA, USA). *, *P* < 0.05 represents a significant difference; **, *P* < 0.01 represents an extremely significant difference.

## Results

### Characterizations of the LL-1 peptide

The physicochemical characteristics of LL-1 were predicted, and the results showed that the average hydropathicity was—0.262; the net charge was 6 + ; The theoretical pI was 11.05; and the molecular weight was 4225.02 Da. To study the *in vitro* cytotoxicity of LL-1, the hemolytic activitie and cytotoxicity of LL-1 were determined in red blood cells of BALB/c mice and PK-15 cells, respectively. The results suggested that the hemolysis rate of LL-1 was only (9.00 ± 1.00) % when red blood cells were treated with 250 μg/mL AC-1 for 1 h (Fig. S1a). The cell survival rate was more than 95% when PK-15 cells were treated with 250 μg/mL LL-1 for 1 h (Fig. S1b). These results showed that LL-1 had low hemolytic and cytotoxic activities.

### Antibacterial effect of LL-1 against *E. coli*

**Fig. 1:**
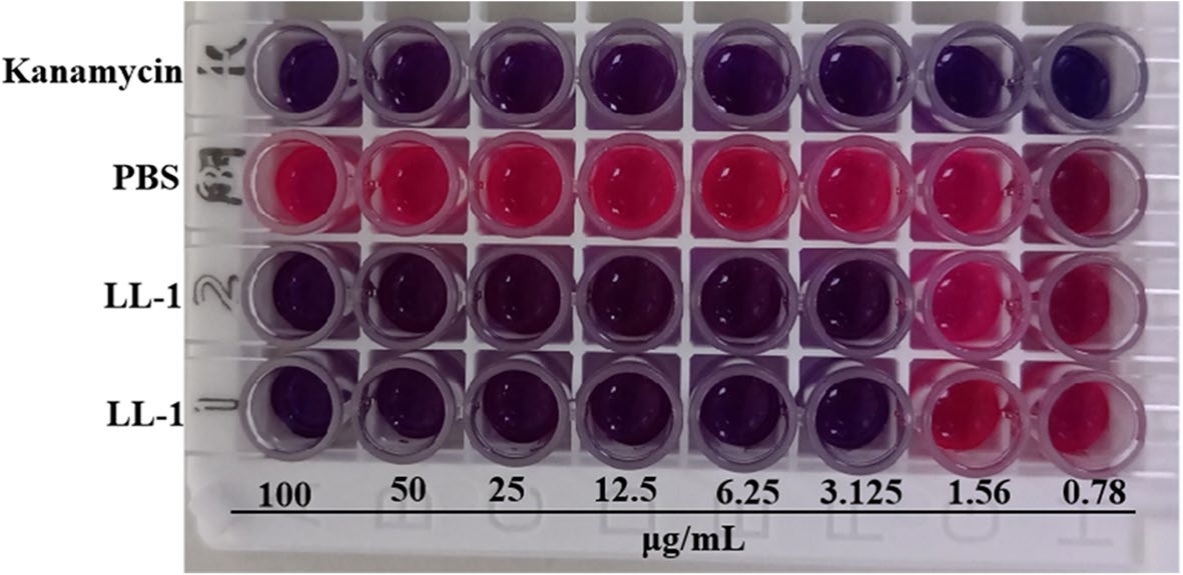
The MIC of LL-1 against *E. coli*

The MIC of LL-1 against *E. coli* was determined by the doubling dilution method. The results suggested that 0.78 µg/mL kanamycin completely inhibited the growth of *E. coli*, and the PBS control showed bacterial growth after treatment at 37 °C for 16 h. The color of the two groups with added LL-1 turned red in the seventh well; thus, the MIC of LL-1 against *E. coli* corresponded to the concentration of the sixth well, which was 3.125 µg/mL, (Fig. 1). The MICs of LL-1 against other bacteria were also determined and are presented in Table S1.

**Fig. 2:**
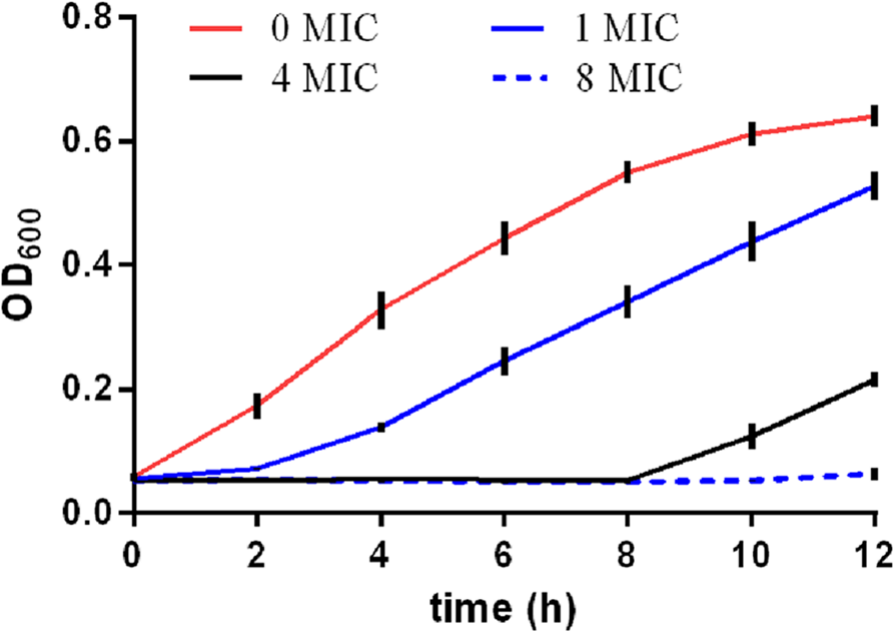
Effects of LL-1 on *E. coli* growth

The antibacterial ability of LL-1 against *E. coli* was evaluated in different growth stages according to the antibacterial curve. Compared with the PBS control, 1 MIC of LL-1 had a good bactericidal effect on *E. coli*, and the antibacterial effect was dose-dependent. Within 12 h, 8 MIC of LL-1 completely inhibited the growth of *E. coli* (Fig. 2).

### Effects of LL-1 on the permeability of *E. coli* cell membrane

**Fig. 3:**
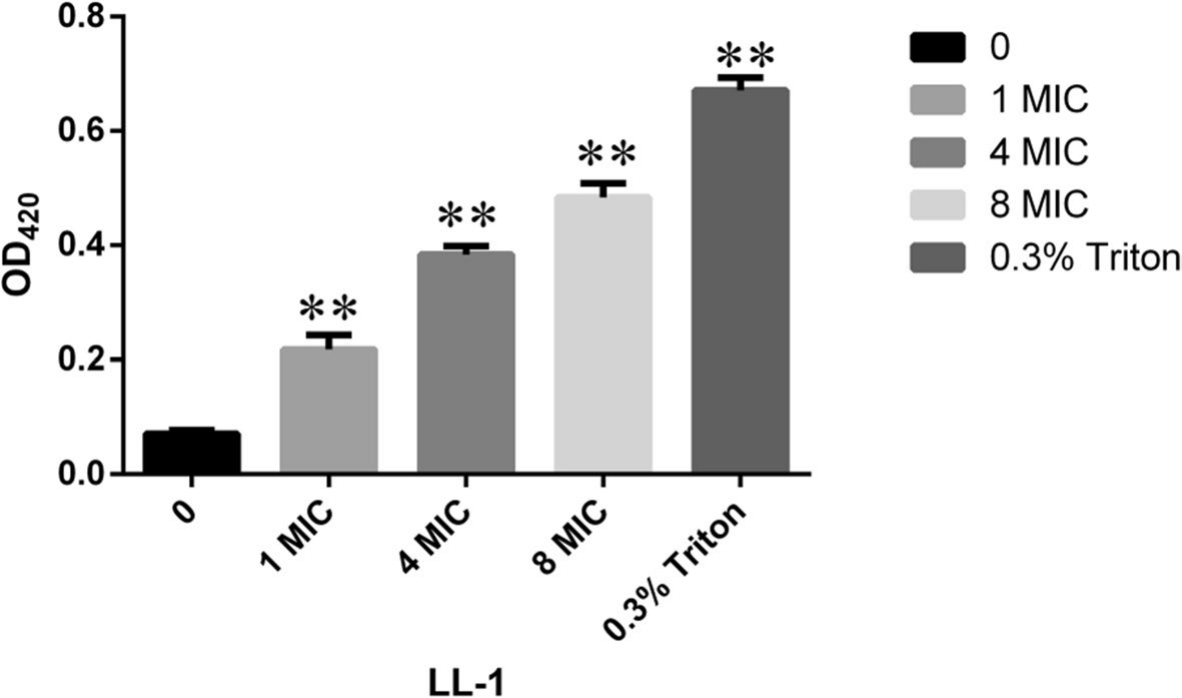
Effects of LL-1 on intracellular β-galactosidase leakage in *E.coli*

**Fig. 4:**
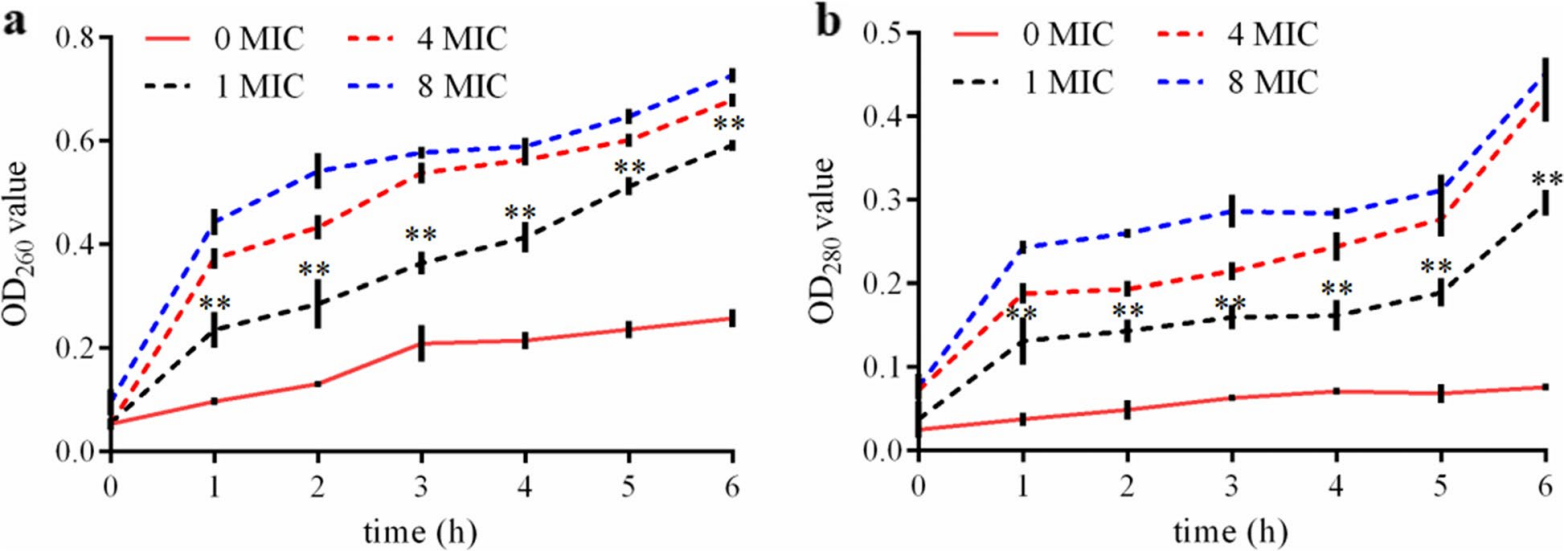
Effects of LL-1 on intracellular nucleic acid (**a**) and protein (**b**) leakage in *E.coli*

The concentration of β-galactosidase in the supernatant of LL-1-treated *E. coli* was determined to evaluate the effects of LL-1 on the permeability of the *E. coli* cell membrane. When *E. coli* was treated with 1 MIC of LL-1 for 1 h, leaked β-galactosidase was significantly higher than that in the control (*P* < 0.01) (Fig. 3). The results indicated that LL-1 led to the leakage of intracellular β-galactosidase in *E. coli* in a dose-dependent manner. In *E. coli* treated with 1 MIC of LL-1, the leakage of nucleic acid and protein was significantly higher than that in the control group at 1, 2, 3, 4, 5, and 6 h post-treatment, respectively (*P* < 0.01), and was dose-dependent (Fig. 4). These results proved that LL-1 increased the permeability of the *E. coli* cell membrane.

### The interaction between LL-1 and *E. coli* DNA

**Fig. 5:**
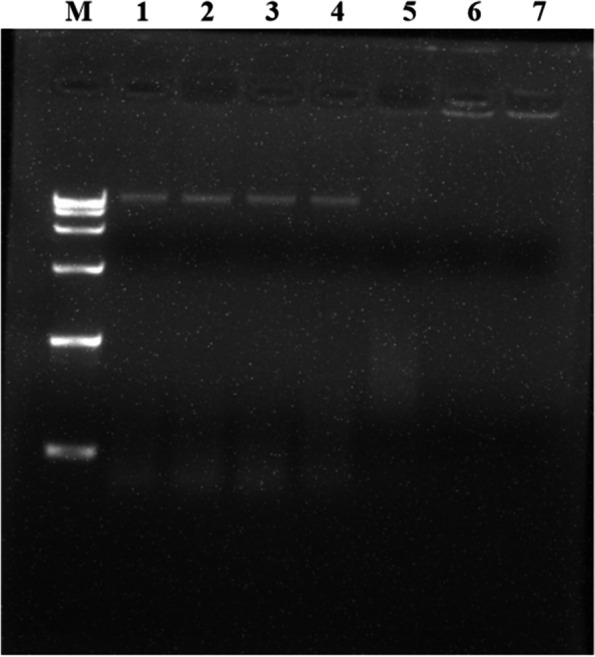
The binding effect of LL-1 and *E. coli* DNA. M: DL-15000 DNA Marker. 1–7: the mass ratio of LL-1 to *E.coli* DNA was 0, 0.25, 0.5, 1, 2.5, 5 and 10, respectively

To preliminarily study the binding effect of LL-1 and *E. coli* DNA, LL-1 at the different concentrations and *E. coli* DNA were incubated together in a constant temperature water bath at 37℃ for 30 min, and then the mixture in each sample was subjected to nucleic acid gel electrophoresis. The results indicated that with increased LL-1 concentration, the brightness of *E. coli* DNA bands gradually decreased, suggesting that LL-1 bound to *E. coli* DNA (Fig. 5).

### Effects of LL-1 on the intracellular ATP of *E. coli*

**Fig. 6:**
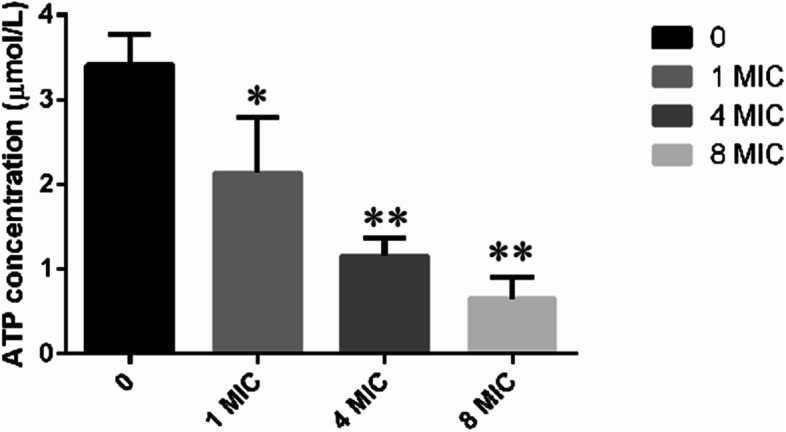
Effects of LL-1 on intracellular ATP concentration in *E.coli*

The changes in intracellular ATP concentration were detected in *E. coli* treated with different concentrations of LL-1 using the ATP Assay Kit. When *E. coli* was treated with 1 MIC of LL-1, the concentration of ATP was significantly lower than that of the control (*P* < 0.05). The concentrations of ATP in the 4 MIC and 8 MIC-treated groups were extremely significantly lower than that of the control (*P* < 0.01) (Fig. 6). These results suggested that LL-1 decreased the intracellular ATP concentration of *E. coli* in a dose-dependent manner.

### Observation of LL-1-treated *E. coli* by transmission and scanning electron microscopy

**Fig. 7:**
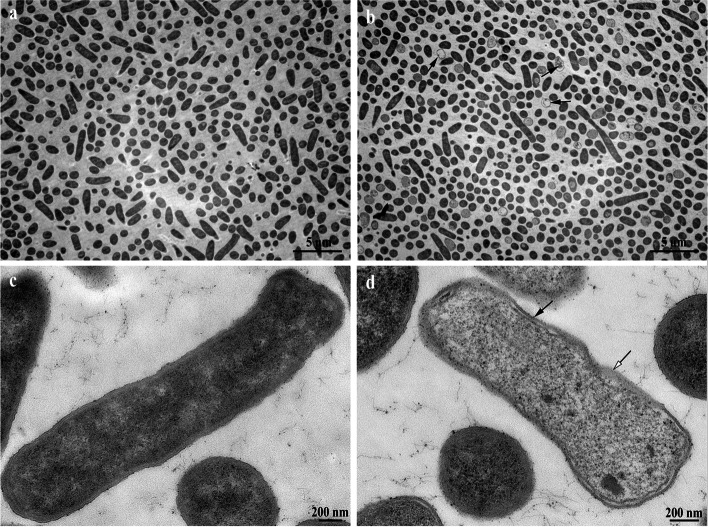
Effect of LL-1 on *E.coli* observed by transmission electron microscopy. **a** PBS-treated *E.coli* observed by transmission electron microscopy. **b** 2 MIC LL-1-treated *E.coli* observed by transmission electron microscopy. Black arrows pointed to dead bacteria. **c** PBS-treated *E.coli* observed by transmission electron microscopy. **d** 2 MIC LL-1-treated *E.coli* observed by transmission electron microscopy. Bacterial plasmolysis occurred at the site indicated by the black arrow. Bacterial membrane lysis occurred at the site indicated by the white arrow

**Fig. 8:**
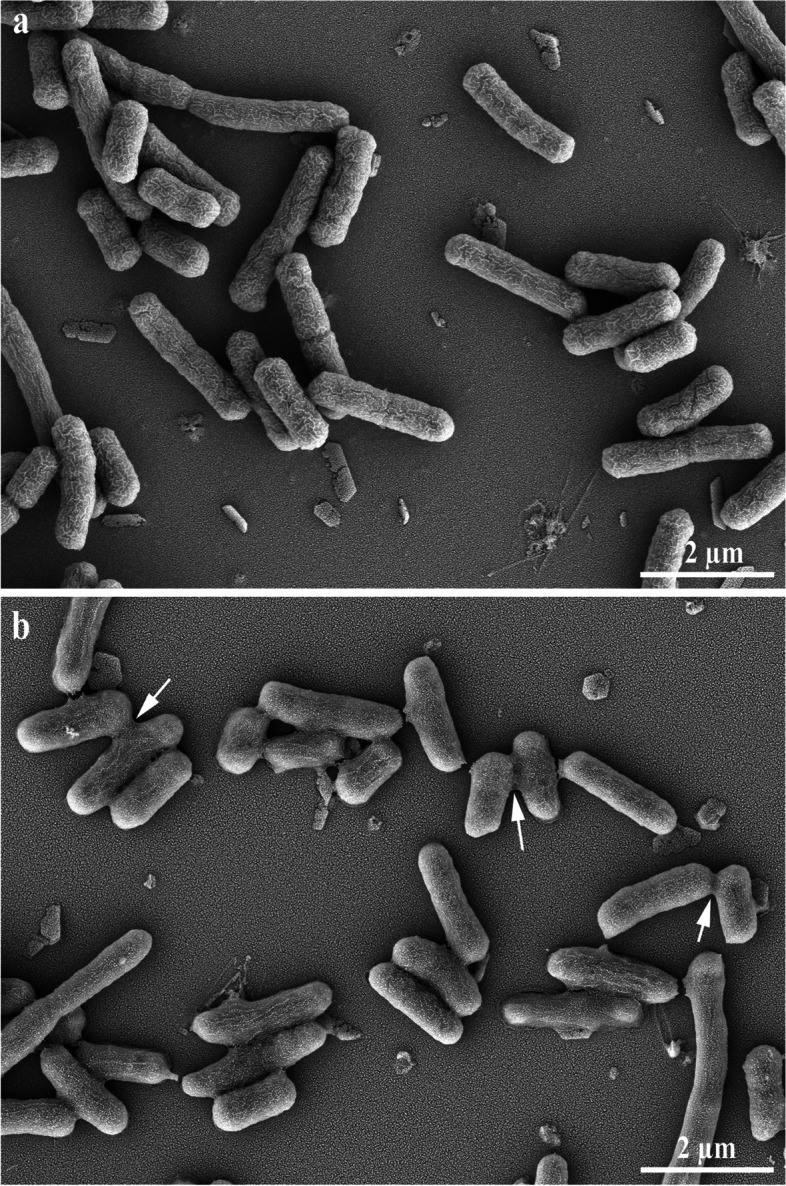
Effect of LL-1 on *E.coli* observed by scanning electron microscopy. **a** PBS-treated *E.coli* observed by scanning electron microscopy. **b** 2 MIC LL-1-treated *E.coli* observed by scanning electron microscopy. Bacterial adhesion occurred at the site indicated by the white arrow

In order to analyse the effect of LL-1 on *E. coli*, LL-1-treated *E. coli* was observed by transmission and scanning electron microscopy. The transmission electron microscopy results showed that compared with the control (Fig. 7a), LL-1 caused bacteria to die, and the bacterial death rate was approximately (15.65 ± 3.56)% (Fig. 7b). compared with the control (Fig. 7c), LL-1 resulted in obvious deformation, bacterial swelling, decreased intracellular electron density, cytoplasmic lysis, and cell membrane damage in *E. coli* (Fig. 7d). The scanning electron microscopy results indicated that compared with the control (Fig. 8a), the surface of *E. coli* in the LL-1 treated sample was rough, the outline of *E. coli* was blurred, and adhesion between *E. coli* was observed (Fig. 8b).

### Effects of LL-1 on *E. coli* determined by PI staining experiment

The results of PI staining experiments indicated that compared with the negative control group, both groups treated with LL-1 and Triton X-100 significantly enhanced the fluorescence intensity, and the fluorescence intensity gradually increased with increased LL-1 concentrations (Fig. S2). PI can not enter into the living cells, but can enter into the dead cells, and PI binding to intracellular nucleic acids will significantly enhance its fluorescence intensity. Therefore, the results suggested that LL-1 had a dose-dependent killing effect on *E. coli*.

## Discussion

Considerable attention has been paid to antimicrobial peptides as an effective alternative to antibiotics [26]. In this study, the MIC of LL-1 on *E. coli* was determined to be 3.125 µg/mL. The MIC of P3 peptide is 12.5 µg/mL against E. coli ATCC 25,922; however, the MIC of its modified peptides JH-0, HJ-1, JH-2, and JH-3 are 6.25, 3.125, 6.25, and 3.125 µg/mL, respectively [18]. The modification of antimicrobial peptide is necessary for future application, and modification could lower the MIC and increase the stability for proteases, pH hydrolysis and temperature [27]. Therefore, the pH hydrolysis and protease stability of LL-1 should be tested in subsequent experiments. The antibacterial curve of LL-1 on *E. coli* was evaluated, and it was found that 8 MIC of LL-1 completely inhibited *E. coli* at 12 h, which indicated that LL-1 had a good *in vitro* antibacterial effect on *E. coli*. Research on the antibacterial mechanism of antimicrobial peptides can lay the foundation for their optimization and clinical application. Therefore, this study preliminarily investigated the antibacterial mode of LL-1 against *E. coli*.

Antibacterial peptides can enter the cell through the cell wall and cell membrane, bind to intracellular DNA and change the conformation of DNA, or degrade DNA, interfere with DNA replication, inhibit RNA transcription, and affect protein synthesis [28–30]. A previous study found that the antimicrobial peptide N4 can penetrate the *E. coli* cell membrane and enter the cell, bind to DNA, destroy the conformation of DNA, inhibit the synthesis of DNA and RNA, affect the bacterial replication cycle and result in the generation of intracellular reactive oxygen species, condense chromatin and cause bacterial apoptosis [19]. In this study, the results of co-incubation of LL-1 with *E. coli* DNA was analysed by nucleic acid gel electrophoresis, and the results showed that LL-1 could bind to *E. coli* DNA. Antimicrobial peptides can bind to lipoteichoic acids of Gram-positive bacteria or lipopolysaccharide of Gram-negative bacteria to affect the permeability of the bacterial cell membrane, resulting in the leakage of intracellular nucleic acid, protein and salt ions, changing the cell membrane potential, and causing bacterial death [28, 31–33]. The antimicrobial peptide temporin-GHa-GHd changed the permeability and morphology of the bacterial cell membrane, making it rough and caused shrinkage, thereby inhibiting *Staphylococcus aureus* (*S. aureus*) [34]. The bovine-derived antimicrobial peptide P3 derivative JH-3 is able to inhibit *Salmonella* [17] and *E. coli* [18] by destroying the cell wall and cell membrane, leading to the leakage of bacterial contents. Johnston et al. [35] proved that telomycin can kill bacteria by inhibiting cardiolipin in bacterial cell membranes. β-galactosidase is a hydrolase located on the bacterial cytoplasmic membrane. Detection of β-galactosidase in the supernatant can evaluate the effects of a drug on the cell membrane permeability [36]. In the present study, LL-1 was able to increase the permeability of *E. coli* cell membrane, resulting in leakage of intracellular β-galactosidase, nucleic acids and proteins. ATP plays an important role in cellular material transport and energy conversion, and changes in its concentration are directly related to cellular energy metabolism; thus, ATP is often used to evaluate the energy metabolism level of bacteria or cells [29, 37]. The study by Liu et al. [20, 38] confirmed that the antimicrobial peptide bacaucin-1 reduces the intracellular ATP of *S. aureus* and leads to cell apoptosis. Similar results were obtained in our study which showed that LL-1 decreased the intracellular ATP concentration of *E. coli* in a dose-dependent manner. The decrease in ATP concentration may be due to the influence of LL-1 on ATP synthesis, or the increase in ATP consumption, or the changes in cell membrane permeability, leading to leakage of ATP, which needs further study. The electron microscopy results showed that LL-1 resulted in obvious deformation, bacterial swelling, decreased intracellular electron density, cytoplasmic lysis, and cell membrane damage in *E. coli*. In the LL-1 treated sample, the bacterial surface became rough, the outline was blurred, and adhesion between *E. coli* was observed.

In conclusion, LL-1 has good antibacterial activity against *E. coli*, mainly by increasing the permeability of *E. coli* cell membrane permeability, which results in the leakage of intracellular contents and affects energy metabolism. This will lay a foundation for further study on the antibacterial mechanism of LL-1 against *E. coli*. The molecular mechanism of the effect of LL-1 on *E. coli* cell membrane permeability requires further investigation.

## Supplementary Information

**Additional file 1: Fig. S1. **Hemolytic and cytotoxic activities of LL-1. a Hemolytic activity of LL-1 determined in BALB/c mice red blood cells. b Cytotoxic activity of LL-1 detected in PK-15 cells.

**Additional file 2: Fig. S2. **Effects of LL-1 on *E. coli *determined by PI staining experiment.

**Additional file 3: Table S1.** Minimum inhibitory concentration (μg/mL) of the LL-1 against microorganisms.

## Data Availability

All data generated or analyzed during this study are included in this published article and its supplementary information files.
