# Supplementary figures and images for: The antimicrobial effect of a novel peptide LL-1 on Escherichia coli by increasing membrane permeability
Source: BMC Microbiol. 2022 Sep 19;22:220. doi: 10.1186/s12866-022-02621-y (PMC9484052; doi:10.1186/s12866-022-02621-y)

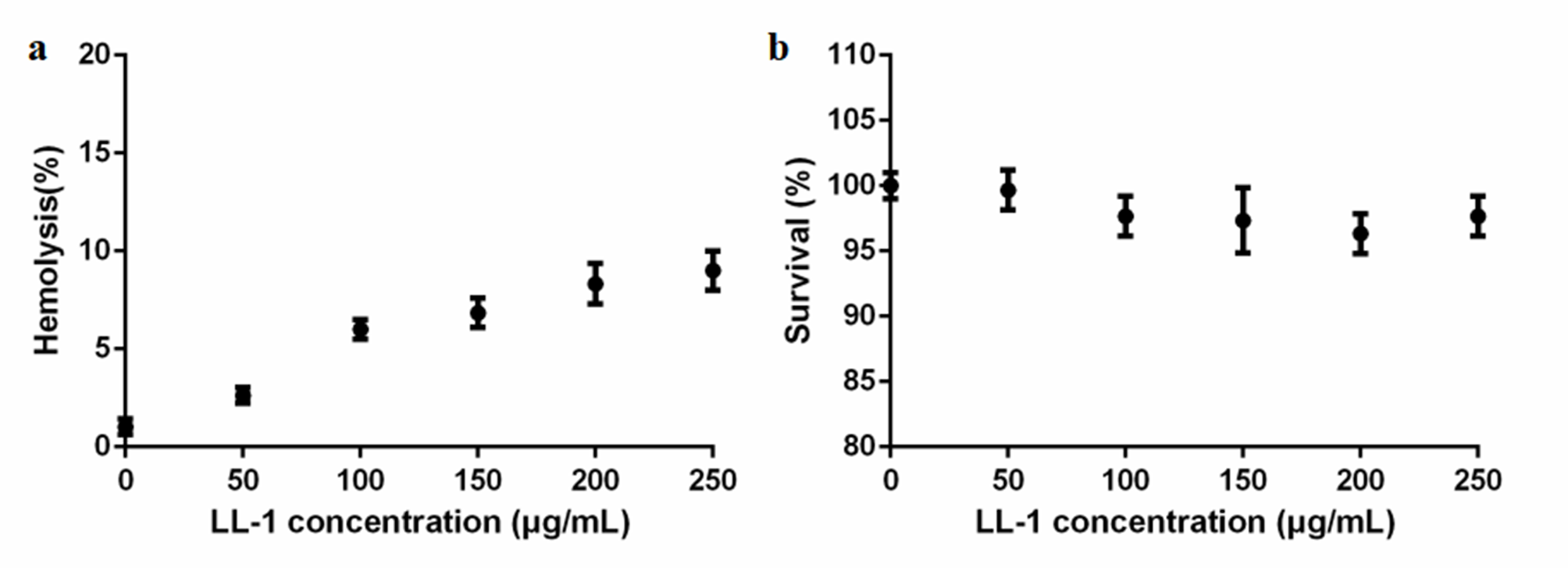

Supplement: Supplementary file 1 — Additional file 1: Fig. S1. Hemolytic and cytotoxic activities of LL-1. a Hemolytic activity of LL-1 determined in BALB/c mice red blood cells. b Cytotoxic activity of LL-1 detected in PK-15 cells. [file 12866_2022_2621_MOESM1_ESM.tif]

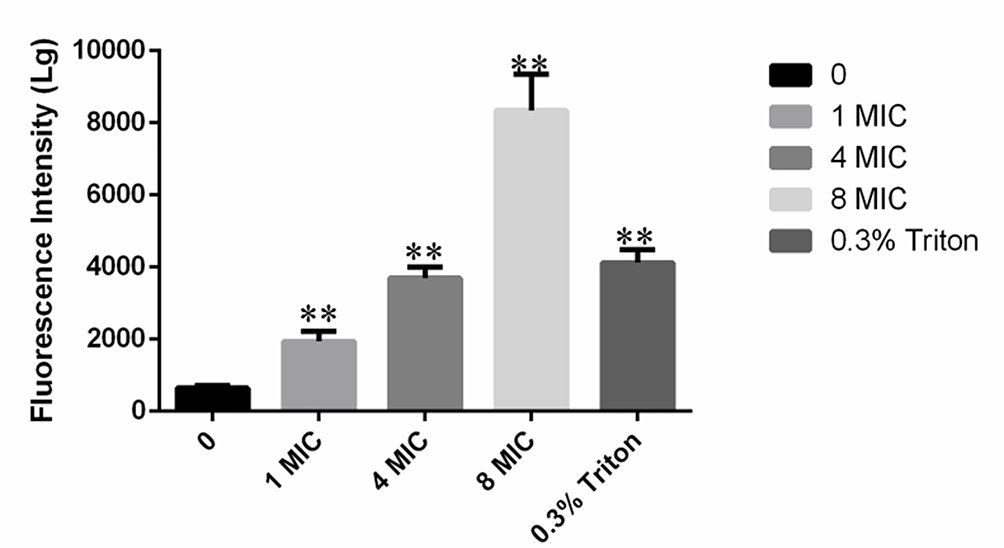

Supplement: Supplementary file 2 — Additional file 2: Fig. S2. Effects of LL-1 on E. coli determined by PI staining experiment. [file 12866_2022_2621_MOESM2_ESM.tif]
