# Supplementary material for: The antimicrobial effect of a novel peptide LL-1 on Escherichia coli by increasing membrane permeability
Source: BMC Microbiol. 2022 Sep 19;22:220. doi: 10.1186/s12866-022-02621-y (PMC9484052; doi:10.1186/s12866-022-02621-y)
Supplement: Supplementary file 3 — Additional file 3: Table S1. Minimum inhibitory concentration (μg/mL) of the LL-1 against microorganisms. [file 12866_2022_2621_MOESM3_ESM.docx]

Table S1 Minimum inhibitory concentration (μg/mL) of the LL-1 against microorganisms

| Microorganisms | MIC (µg/mL) |
| --- | --- |
| *Escherichia coli* ATCC25922 | 3.125 |
| *Escherichia coli* clinical strain | 12.5 |
| *Salmonella* ATCC13076 | 3.125 |
| *Salmonella* clinical strain | 25 |
| *Klebsiella pneumonia* ATCC27853 | 12.5 |
| *Pseudomonas aeruginosa* ATCC700603 | 12.5 |
| *Bacillus cereus* ATCC11778 | >250.00 |
| *Staphylococcus aureus* ATCC29213 | >250.00 |
